# Factors Associated with Turnover Intentions of Nurses Working in Japanese Hospitals Admitting COVID-19 Patients

**DOI:** 10.3390/nursrep13020069

**Published:** 2023-05-20

**Authors:** Yoshiko Kitamura, Hisao Nakai

**Affiliations:** 1 School of Nursing, Kanazawa Medical University, 1-1 Uchinada, Kahoku 920-0265, Ishikawa, Japan; 2 Faculty of Nursing, University of Kochi, 2751-1 Ike, Kochi City 781-8515, Kochi, Japan; nakai_hisao@cc.u-kochi.ac.jp

**Keywords:** COVID-19 pandemic, turnover intention, nurse, cross-sectional study, Japan

## Abstract

Three years after the outbreak of the coronavirus disease (COVID-19) pandemic, turnover among frontline nurses has increased. The participants of this study were nurses at two general hospitals in Ishikawa, Japan, receiving COVID-19 patients. An original self-report questionnaire was created based on previous research. The questionnaire was distributed to 400 nurses, and responses were received from 227 nurses (response rate: 56.8%). The factors influencing turnover intention at the facilities were having less time to relax (odds ratio [OR]: 2.88, 95% confidence interval [CI]: 1.12–7.41) and wanting to receive counseling (OR: 5.21, 95% CI: 1.30–20.91). As a strategy to prevent turnover, nurse managers should provide opportunities for nurses to receive counseling during normal working hours and pay particular attention to changes in nurses’ daily lives, such as changes in the time available for relaxation.

## 1. Introduction

The first case of coronavirus disease (COVID-19) was reported in Wuhan, China, in December 2019 [1]. Three years have passed since the World Health Organization (WHO) declared a public health emergency of international concern on 30 January 2020 [2]. The WHO has worked with public health agencies globally to address this issue. In addition to vaccination, public health departments have urged the citizens of their countries to practice hand hygiene, social distancing, and quarantine to prevent the spread of the virus and secondary infections [3,4,5]. Frontline hospitals hosting COVID-19 implemented a blanket approach of active and enhanced laboratory surveillance, early airborne isolation, rapid molecular diagnostic testing, and contact tracing of unprotected and exposed healthcare workers in the hospitals [6,7,8]. However, complete containment of COVID-19 has yet to be achieved. According to the WHO, 660 million infections, including 6.7 million deaths, were reported in the period ending 20 January 2023 [2,3].

The COVID-19 pandemic has had a substantial effect on the physical and mental health of healthcare workers who treat patients [8,9]. Hospital medical staff receiving COVID-19 patients constantly experience emotional distress, and many report a high psychological burden [10,11,12]. For instance, Spanish nursing home workers reported high workloads, social pressures from work, contact with affected people who were ill, and fear of contagion [13]. The effects of psychological distress on frontline nurses are also severe [14]. Nurses in Portugal were exposed to higher rates of depression, anxiety, and stress compared to the general population of Portugal [15]. A 2020 study in New York City reported that nurses/advanced practice providers had higher rates of acute stress and depression than primary care physicians [16]. A study of frontline nurses during an outbreak in Chongqing, China, reported that approximately 43% of nurses complained of physical discomfort and had depression, anxiety, and suicidal thoughts [17].

The COVID-19 pandemic has increased the demand for nurses and increased their workload. Additionally, there has been a substantial increase in the intention to resign among nurses dealing with patients on the COVID-19 frontline [18,19,20,21]. Nurses were nearly four times more likely than other healthcare providers to consider leaving their jobs because of COVID-19 [22], and nurses in hospitals receiving COVID-19 patients had higher occupational stress and greater intention to leave the nursing field than nurses in general hospitals [23]. A literature review of nurses’ intention to resign because of COVID-19 highlighted the high turnover rate of nurses after the pandemic compared with before [24]. Japan is no exception; many nurses on the frontline of the pandemic have left the workforce. A survey by the Japanese Nursing Association reported a higher turnover rate at designated medical institutions for infectious diseases than at general hospitals [25]. A survey of hospitals accepting COVID-19 patients in Japan also showed that the increase in COVID-19 patients had a negative effect on nurses’ mental health and intention to resign [26]. Japanese critical care professionals have suggested that intensive care unit nurses may experience increased burnout and turnover intention because they routinely bear the responsibility for healthcare during pandemics and are exposed to infection through their close contact with patients [27]. It is therefore important to identify factors that affect turnover intentions, focusing on the workload of frontline nurses in hospitals receiving COVID-19 patients.

As the largest group of healthcare providers who spend the most time with patients, nurses have been most affected by the pandemic [24]. The global nursing shortage has long been recognized as an important policy concern [28], and the COVID-19 pandemic has further raised concerns about the shortage of nurses. The turnover of nurses specializing in critical care for COVID-19 patients will affect the acute care of non-COVID-19 patients. Furthermore, the turnover of frontline nurses will have a substantial impact on the continuity of healthcare in hospitals, considering that nurses specializing in critical care must undergo several years of post-licensure training.

Continuing research on turnover in nurses has identified complex relationships among organizational and individual factors [29]. However, it has not considered changes in nurses’ work and daily lives caused by the COVID-19 pandemic. Therefore, this study aimed to identify factors associated with turnover intention among nurses working in hospitals that routinely admit COVID-19 patients in Japan, 3 years after the COVID-19 pandemic. The need to manage COVID-19 infections will persist for some time. Identifying factors associated with the turnover intentions of nurses working with COVID-19 patients may contribute to human resource-related risk assessments undertaken by nursing management in this context.

## 2. Materials and Methods

Our manuscript is compliant with the “Strengthening the Reporting of Observational studies in Epidemiology” (STROBE) guidelines [30].

### 2.1. Questionnaire Survey

We conducted a web-based online cross-sectional survey of 400 nurses working at two medical institutions in Ishikawa Prefecture, Japan. These two medical institutions accept patients with COVID-19 infection at the request of Ishikawa Prefecture.

This research used survey items from our previous study, “Factors Affecting Nurses’ Internal Transfer Intentions after the Introduction of COVID-19-Related Family Visiting Restrictions [31]”. In Japan, the turnover of nurses on the frontline has increased because of the COVID-19 pandemic. Drawing on our awareness of this problem, we conducted a further survey of nurses at hospitals accepting patients with COVID-19. Nursing administrators were excluded from the study because their role is to improve nurses’ working environment. The survey items were based on interviews with four nurses about changes in their work owing to the COVID-19 pandemic. In addition, we referred to reports by Matsuo et al. of St Luke’s International Hospital in Japan [32] and Awano et al. [33] of the Japanese Red Cross Medical Center. The original questionnaire was designed as a self-administered, anonymous survey, based on these two reports. All questions were tailored to the context of the survey. Surveys were generated using SurveyMonkey from Momentive Global Inc. (San Mateo, CA, USA). SurveyMonkey is a web-based survey application. A link to the questionnaire platform was sent to participants via nursing administrators. The survey period was from 15 January 2022 to 4 March 2022.

### 2.2. Survey Content

#### 2.2.1. Participant Attributes and Years of Experience

We asked participants about their ages (in their 20 s, 30 s, 40 s, 50 s, or 60 s), sex, and years of experience.

#### 2.2.2. Pandemic-Related Changes in Nurses’ Work Content

Questions regarding visitor guidance, assisting with visits using the patient’s smartphone, assisting the physician with online explanations to family members, delegating shopping, delegating phone calls, transporting patients who transferred to another hospital or to a nursing home, and transferring the patient to the hospital entrance upon discharge were responded to using “increased significantly”, “increased”, “no change”, “decreased”, or “decreased significantly”.

#### 2.2.3. Pandemic-Related Changes in Nurses’ Working Hours, Number of Days Off, and Sleeping Hours

Questions regarding working hours, number of days off, and sleeping hours were responded to using “increased significantly”, “increased”, “no change”, “decreased”, or “decreased significantly”.

#### 2.2.4. Pandemic-Related Changes in Nurses’ Daily Lives

Questions regarding unhealthy diet, sleeping less, waking up more in the middle of the night, smoking more, and having less time to relax were responded to using “strongly agree”, “agree”, “disagree”, or “strongly disagree”.

#### 2.2.5. Support Needed for Nurses Working during the COVID-19 Pandemic

Participants were asked if they needed more nurses, more nursing assistants, appreciation, respect, benefits, counseling, workload reduction, infection prevention education, and childcare support. Possible responses for each item were “strongly agree”, “agree”, “disagree”, or “strongly disagree”.

#### 2.2.6. Turnover Intention

Nurses working in Japanese hospitals accepting COVID-19 patients were asked about their intention to resign; possible responses were “yes” or “no”.

### 2.3. Analytical Method

The analysis was based on reports examining burnout and turnover intention among Romanian nurses during the COVID-19 pandemic [34]. The chronological age of the nurses was divided into two categories: 20 s, and 30 years or older. Years of nursing experience has been reported as a variable that influences turnover intention [27,35], with turnover intention in the COVID-19 pandemic higher for nurses with fewer years of experience [24,36]. The present study investigated the distribution of nurses’ years of experience during the COVID-19 pandemic. We calculated the median distribution of years of experience and used that number to divide experience into two categories: less than 15 years and more than 15 years.

Pandemic-related changes in nurses’ work content, working hours, number of days off, and sleeping hours were categorized as “increased” for “increased significantly/increased”, and other responses were categorized as “others”.

To analyze pandemic-related changes in nurses’ daily lives and participants’ perception of the support they needed for working during the COVID-19 pandemic, responses of “strongly agree/agree” were categorized as “agree”, and responses of “disagree/strongly disagree” were categorized as “disagree”.

### 2.4. Statistical Analyses

We conducted *χ*^2^ Fisher direct probability tests on the association between turnover intention and participant attributes and years of experience; pandemic-related changes in work content, working hours, number of days off, sleeping hours, daily lives, and perceived support needed for nurses working during the COVID-19 pandemic.

To evaluate the effects on turnover intention of participant attributes and years of experience, pandemic-related changes in work content, working hours, number of days off, sleeping hours, daily lives and perceived support needed during the COVID-19 pandemic, we performed a univariate analysis with turnover intention as the dependent variable and sex, age, and years of experience as covariates. The responses “Difficulty falling asleep”, “I have experienced increased night waking”, “Less time to relax”, and “I want to receive counseling” were shown to have a significant probability value of <5%. Thus, these were forced into the model and a stepwise binomial logistic regression analysis was conducted.

The variables used for analysis were entered after checking for multicollinearity (variance inflation factor ≥10), and the statistical significance level was 5%. All data entry, tabulation, and statistical analysis were performed using SPSS Ver27 from IBM Corp. (Armonk, NY, USA).

### 2.5. Ethical Approval

This study was conducted with the consent of the Academic Research Ethics Review Board of the first author’s university (No. I673) and was conducted in accordance with the 1995 Declaration of Helsinki (revised in Seoul, 2008). Nursing administrators at two hospitals were requested to cooperate with the study by telephone and email. A letter of informed consent was distributed to the participants via email, informing them of the purpose and importance of the study, the survey methodology, that participation was voluntary, that participants’ responses were anonymous, so that no individuals would be identified by answering the survey, and that completion of the questionnaire implied their consent.

## 3. Results

### 3.1. Relationship of Turnover Intention with Nurses’ Work Burden, Work Changes, and Changes in Daily Life

Of the total sample of 400 nurses, 227 responded to the survey (56.8% response rate). Of these, 134 (59.0%) responded to all the survey items and their data were included in the analyses. Four (3.0%) participants were male, 130 (97.0%) were female, with 27 (20.1%) in their 20 s and 107 (79.9%) ≥30 years old. The median (range of) years of experience was 15 years (1–40): <15 years, 63 participants (47.0%); ≥15 years, 71 participants (53.0%) (Table 1).

In terms of changes in nurses’ work content after the COVID-19 pandemic, “increased” was the most common response for “Delegating phone calls” (*n* = 107, 79.9%), “Visitor guidance”, “Transferring the patient to the hospital entrance upon discharge” (*n* = 96, 71.6%), and “Delegating shopping” (*n* = 95, 70.9%).

Regarding negative pandemic-related changes in working hours, number of days off, and sleeping hours, “agree” was the most common response for “Working hours” (*n* = 66, 49.3%), “Sleeping hours” (*n* = 6, 4.5%), and “Number of days off” (*n* = 2, 1.5%).

In terms of pandemic-related changes in nurses’ daily lives, “agree” was the most common response to “Less time to relax” (*n* = 89, 66.4%), “I have experienced increased night waking” (*n* = 54, 40.3%), and “Difficulty falling asleep” (*n* = 49, 36.6%).

In terms of support needed for nurses working during the COVID-19 pandemic, “agree” was the most common response to “Need more nurses” (*n* = 131, 97.8%), “Need more nursing assistants” (*n* = 129, 96.3%), and “Wants to have a pay raise” (*n* = 127, 94.8%).

The number of respondents who answered “Yes” to the question about turnover intention was 85 (63.4%) (Table 1).

### 3.2. Analysis of Factors Associated with Turnover Intentions of Nurses Caring for COVID-19 Patients

Regarding the relationship between turnover intention and participant attributes and years of experience, pandemic-related changes in nurses’ work content, working hours, number of days off, sleeping hours, daily lives, and perceived support needed for nurses working during the COVID-19 pandemic, the results showed that the following categories of nurses had higher turnover intention: aged ≥30 years (*n* = 62, 57.9%) (*p* = 0.009); having ≥15 years of experience (*n* = 37, 52.1%) (*p* = 0.004); and those agreeing to “Difficulty falling asleep” (*n* = 41, 83.7%) (*p* < 0.001), “I have experienced increased night waking” (*n* = 43, 79.6%) (*p* = 0.001), “Less time to relax” (*n* = 67, 75.3%) (*p* < 0.001), “Wants to have a pay raise” (*n* = 84, 66.1%) (*p* = 0.010), “Want to receive counseling” (*n* = 21, 87.5%) (*p* = 0.007), and “Want to reduce my workload” (*n* = 83, 68.0%) (*p* = 0.001) (Table 1).Binomial logistic regression analysis was used to examine factors associated with turnover intention among nurses working in Japanese hospitals accepting COVID-19 patients. The identified factors were “Less time to relax” (odds ratio [OR] 2.875, 95% confidence interval [CI]: 1.116–7.408) and “Want to receive counseling” (OR 5.212, 95% CI: 1.299–20.914) (Table 2).

## 4. Discussion

The purpose of our study was to identify factors associated with turnover intention among nurses working in hospitals accepting COVID-19 patients during the pandemic. Of the participants, 63.4% expressed a turnover intention because of the COVID-19 pandemic. Nurses who indicated they had less time to relax and expressed a wish to receive counseling were more likely to express turnover intention than those with different responses. Break length and the frequency of social breaks are not strong predictors of nurse turnover [37]. The implementation of regularly scheduled breaks may be an effective intervention to improve nurse retention [38]. Many studies have illuminated the importance of proactive counseling as a countermeasure to nurses’ psychological distress, burnout, and turnover during the COVID-19 pandemic [16,39,40,41,42,43,44]. Our results are consistent with these previous reports and indicate the need for nursing managers to be attentive to the stress and psychological distress of frontline nurses, and to ensure their availability for consultation any time.

Perceived reduced relaxation time outside of work may be a predictor of turnover intention in frontline nurses. Intensive care unit nurses in charge of COVID-19 patients should relax outside of work hours to reduce perceived stress [45]. Nurses who engage in relaxation activities after work hours report less anxiety than those who do not [46]. However, some nurses found it difficult to relax during the pandemic period, even when they engaged in their usual relaxation activities [47]. A survey of nursing students during the COVID-19 pandemic indicated that irritability, uncontrollable worry, and trouble relaxing characterized the onset of anxiety and depression [48]. In other words, during a pandemic, nurses experience psychological distress, such as anxiety and depression, prior to turnover intention. Having less time to relax outside of work hours may be an important factor in preventing turnover. Effective interventions have been developed to improve the well-being of nurses by introducing short breaks [49,50]. Nursing managers may need to pay attention to the psychological distress of frontline nurses and consider whether nurses relax sufficiently outside of work hours.

There are several study limitations. Participants worked in a small number of hospitals in a limited geographic area, and were drawn from only general hospitals. In addition, the data available for analysis were limited: only 33.5% of the 227 participants (response rate: 56.8%) were included in the analysis. Participants in this study were asked to complete the survey through their director of nursing. The timing of the survey may have meant that some participants were intending to leave their jobs but unable to respond to this effect. This was a cross-sectional study motivated by the increased turnover rate of nurses caring for COVID-19 patients on the frontline of the COVID-19 pandemic. Therefore, it may have included nurses who intended to resign prior to the COVID-19 pandemic. A recent report on nurses’ turnover intentions has shown that work–family [51] conflicts and job autonomy [52] are involved. However, these variables were not investigated in this study, so we could not determine whether they affected turnover intention. The use of retrospective, change-oriented response formats in this survey meant that responses may have been subject to recall bias. In addition, because this was a cross-sectional study it was not possible to establish a causal relationship between the variables under study.

## 5. Conclusions

To prevent turnover among frontline nurses during pandemics, it is important to be attentive to their psychological distress and provide them with access to counseling, as noted in previous studies. In addition, attention should be paid to whether nurses have reduced time for relaxation outside of work hours. Increases were most frequently reported for the workload activities of delegating phone calls, visitor guidance, and transferring patients to the hospital entrance upon discharge. Although these tasks include aspects that can be handled by nursing assistants, our findings suggest that their performance had become an additional work burden for nurses. During the COVID-19 pandemic, nursing administrators were encouraged to manage increased workloads by reallocating tasks to nurses and nursing assistants. Nursing managers are recommended to implement measures such as establishing a system where counseling is available, encouraging more relaxing activities outside of work hours, and interviewing nurses about whether they are relaxing. On the basis of our findings, we recommend that nursing managers pay attention to pandemic-related changes in nurses’ workloads and increased work burden, and create an environment where nurses can better focus on their work. In addition, nursing managers should monitor nurses’ psychological distress more closely and take prompt mitigation measures, such as shortening exposure time to COVID-19 patients and implementing job rotation. Future research should investigate the issues of increased workload, burden, and psychological distress in frontline nurses. Research is also needed to examine the effect of an organized counseling system and relaxation time for nurses on preventing nurse turnover.

## Figures and Tables

**Table 1:** Relationship between turnover intention and nurses’ participant attributes and years of experience; pandemic-related changes in nurses’ work content, working hours, number of days off, sleeping hours, daily lives, and perceived support needed (*N* = 134).

Item		Category	Total		Turnover Intention				*p*-Value
					Yes		No		
			*N*	(%)	*N*	(%)	*N*	(%)	
Nurses’ Participant attributes and years of experience									
	Sex	Male	4	(3.0)	2	(50.0)	2	(50.0)	0.623 ^(b)^
		Female	130	(97.0)	83	(63.8)	47	(36.2)	
	Chronological age	20 s	27	(20.1)	23	(85.2)	4	(14.8)	0.009 ^(a)^
		≥30 years	107	(79.9)	62	(57.9)	45	(42.1)	
	Years of experience	<15	63	(47.0)	48	(76.2)	15	(23.8)	0.004 ^(a)^
		≥15	71	(53.0)	37	(52.1)	34	(47.9)	
Changes in Nurses’ Work Contents Due to COVID-19									
	Visitor guidance	Increased	96	(71.6)	64	(66.7)	32	(33.3)	0.217 ^(a)^
		Others	38	(28.4)	21	(55.3)	17	(44.7)	
	Assisting with visits using the patient’s smartphone	Increased	90	(67.2)	56	(62.2)	34	(37.8)	0.677 ^(a)^
		Others	44	(32.8)	29	(65.9)	15	(34.1)	
	Assisting the physician with online explanations to family members	Increased	80	(59.7)	50	(62.5)	30	(37.5)	0.785 ^(a)^
		Others	54	(40.3)	35	(64.8)	19	(35.2)	
	Delegating shopping	Increased	95	(70.9)	58	(61.1)	37	(38.9)	0.372 ^(a)^
		Others	39	(29.1)	27	(69.2)	12	(30.8)	
	Delegating phone calls	Increased	107	(79.9)	67	(62.6)	40	(37.4)	0.696 ^(a)^
		Others	27	(20.1)	18	(66.7)	9	(33.3)	
	Transport of patients in transfer to other hospital or nursing home	Increased	48	(35.8)	28	(58.3)	20	(41.7)	0.360 ^(a)^
		Others	86	(64.2)	57	(66.3)	29	(33.7)	
	Transferring the patient to the hospital entrance upon discharge	Increased	96	(71.6)	60	(62.5)	36	(37.5)	0.722 ^(a)^
		Others	38	(28.4)	25	(65.8)	13	(34.2)	
Change in working hours, number of days off, and sleeping hours for nurses due to COVID-19									
	Working hours	Agree	66	(49.3)	46	(69.7)	20	(30.3)	0.138 ^(a)^
		Disagree	68	(50.7)	39	(57.4)	29	(42.6)	
	Number of days off	Agree	2	(1.5)	2	(100.0)	0	(0.0)	0.533 ^(b)^
		Disagree	132	(98.5)	83	(62.9)	49	(37.1)	
	Sleeping hours	Agree	6	(4.5)	5	(83.3)	1	(16.7)	0.415 ^(b)^
		Disagree	128	(95.5)	80	(62.5)	48	(37.5)	
Changes in nurses’ daily lives due to COVID-19									
	Unhealthy eating	Agree	43	(32.1)	31	(72.1)	12	(27.9)	0.152 ^(a)^
		Disagree	91	(67.9)	54	(59.3)	37	(40.7)	
	Difficulty falling asleep	Agree	49	(36.6)	41	(83.7)	8	(16.3)	0.000 ^(a)^
		Disagree	85	(63.4)	44	(51.8)	41	(48.2)	
	Increased number of awakenings during the night	Agree	54	(40.3)	43	(79.6)	11	(20.4)	0.001 ^(a)^
		Disagree	80	(59.7)	42	(52.5)	38	(47.5)	
	Increased smoking	Agree	3	(2.2)	1	(33.3)	2	(66.7)	0.554 ^(b)^
		Disagree	131	(97.8)	84	(64.1)	47	(35.9)	
	Less time to relax	Agree	89	(66.4)	67	(75.3)	22	(24.7)	0.000 ^(a)^
		Disagree	45	(33.6)	18	(40.0)	27	(60.0)	
Support needed for nurses working during the COVID-19 pandemic									
	Need more nurses	Agree	131	(97.8)	84	(64.1)	47	(35.9)	0.554 ^(b)^
		Disagree	3	(2.2)	1	(33.3)	2	(66.7)	
	Needs more nursing assistants	Agree	129	(96.3)	83	(64.3)	46	(35.7)	0.355 ^(b)^
		Disagree	5	(3.7)	2	(40.0)	3	(60.0)	
	Wants to be thanked	Agree	79	(59.0)	54	(68.4)	25	(31.6)	0.156 ^(a)^
		Disagree	55	(41.0)	31	(56.4)	24	(43.6)	
	Wants to be respected	Agree	46	(34.3)	29	(63.0)	17	(37.0)	0.946 ^(a)^
		Disagree	88	(65.7)	56	(63.6)	32	(36.4)	
	Wants to have a pay raise	Agree	127	(94.8)	84	(66.1)	43	(33.9)	0.010 ^(b)^
		Disagree	7	(5.2)	1	(14.3)	6	(85.7)	
	Want to receive counseling	Agree	24	(17.9)	21	(87.5)	3	(12.5)	0.007 ^(a)^
		Disagree	110	(82.1)	64	(58.2)	46	(41.8)	
	Want to reduce my workload	Agree	122	(91.0)	83	(68.0)	39	(32.0)	0.001 ^(b)^
		Disagree	12	(9.0)	2	(16.7)	10	(83.3)	
	Wants to receive infection prevention education	Agree	87	(64.9)	58	(66.7)	29	(33.3)	0.290 ^(a)^
		Disagree	47	(35.1)	27	(57.4)	20	(42.6)	
	Wants to receive parenting support	Agree	73	(54.5)	44	(60.3)	29	(39.7)	0.406 ^(a)^
		Disagree	61	(45.5)	41	(67.2)	20	(32.8)	

^(a)^ *χ*^2^ test, ^(b)^ Fisher’s exact test.

**Table 2:** Relationship between turnover intention and sex, chronological age, years of experience, trouble falling asleep, increased night waking, less time to relax, and desire for counseling.

Item	Category	OR	95% CI		*p*-Value
			Lower Limit	Upper Limit	
Sex	Female/male	0.681	0.079	5.886	0.727
Chronological age	≥30/20 s	3.065	0.728	12.904	0.127
Years of experience	≥15/<15	2.300	0.882	6.001	0.089
Difficulty falling asleep	Agree/disagree	1.486	0.459	4.818	0.509
I have experienced increased night waking	Agree/disagree	2.193	0.727	6.612	0.163
Less time to relax	Agree/disagree	2.875	1.116	7.408	0.029
Want to receive counseling	Agree/disagree	5.212	1.299	20.914	0.020

Binomial logistic regression analysis. Abbreviations: CI: confidence interval; OR: odds ratio.

## Data Availability

The data that support the findings of this study are available upon request to the corresponding authors.
